# The effect of the *TM6SF2* E167K variant on liver steatosis and fibrosis in patients with chronic hepatitis C: a meta-analysis

**DOI:** 10.1038/s41598-017-09548-9

**Published:** 2017-08-24

**Authors:** Zhengtao Liu, Shuping Que, Lin Zhou, Shusen Zheng, Stefano Romeo, Adil Mardinoglu, Luca Valenti

**Affiliations:** 1 Science for Life Laboratory, KTH - Royal Institute of Technology, SE-171 21 Stockholm, Sweden; 2 Key Laboratory of Combined Multi-Organ Transplantation, Ministry of Public Health, First Affiliated Hospital, School of Medicine, Zhejiang University, Hangzhou, 310003 China; 3 Department of Pediatrics, Women and children’s hospital of Guangxi, Nanning, 530005 Guangxi province China; 4 Sahlgrenska Center for Cardiovascular and Metabolic Research, Wallenberg Laboratory, Department of Molecular and Clinical Medicine, Department of Cardiology, University of Gothenburg, Gothenburg, SE-413 45 Sweden; 5 Clinical Nutrition Unit, Department of Medical and Surgical Sciences, University Magna Graecia, Catanzaro, 88100 Italy; 6 Department of Biology and Biological Engineering, Chalmers University of Technology, Gothenburg, Sweden; 7 Department of Pathophysiology and Transplantation, Università degli Studi di Milano, Milan, Italy; 8 Internal Medicine, Fondazione IRCCS Ca’ Granda Ospedale Policlinico Milano, Milan, Italy

## Abstract

The impact of *Transmembrane 6 superfamily member 2* (*TM6SF2*) E167K variant, which causes hepatocellular fat retention by altering lipoprotein secretion, on liver damage and metabolic traits in chronic hepatitis C patients is still debated. We performed a systematic review and meta-analysis to clarify this relationship. Four studies with a total of 4325 patients were included. The risk of histologically-determined advanced steatosis, fibrosis, and cirrhosis (but not of severe inflammation) were increased in carriers of the *TM6SF2* variant (P < 0.05). Unlike the inconsistent association with steatosis severity, due to the confounding effect of infection by the genotype-3 hepatitis C virus, the *TM6SF2* variant was robustly associated with advanced fibrosis (OR = 1.07; 95% confidence interval [CI] = 1.01–1.14) and in particular with cirrhosis (OR = 2.05; 95% CI = 1.39–3.02). Regarding metabolic features, individuals positive for the *TM6SF2* variant exhibited 5.8–12.0% lower levels of circulating triglycerides and non-HDL cholesterol (*P* < 0.05). Carriers of the variant were leaner, but there was high heterogeneity across studies (I^2^ = 97.2%). No significant association was observed between the *TM6SF2* variant and insulin resistance or hepatitis C viral load (both *P* > 0.05). In conclusion, the *TM6SF2* E167K variant promotes the development of steatosis, fibrosis and cirrhosis in patients with chronic hepatitis C. Conversely, this variant reduces circulating atherogenic lipid fractions.

## Introduction

Despite the advent of highly effective direct antiviral agents^1^, hepatitis C virus (HCV) infection still affects more than 130 million individuals worldwide and accounts for a large proportion of liver-related mortality. However, in patients with chronic hepatitis C (CHC) there is a wide inter-individual variability in the susceptibility to develop of progressive liver disease, which may lead to cirrhosis and hepatocellular carcinoma^2^.

Steatosis, defined as hepatocellular fat accumulation exceeding 5% of liver weight, is a typical histological feature of CHC, which is associated with higher likelihood of progression of liver damage and fibrosis^3, 4^. Both viral factors, e.g., infection by HCV-genotype 3 (G3), and host factors, including overweight, type 2 diabetes and at risk alcohol intake, contribute to the development of steatosis in patients with CHC^5–7^.

Inherited host factors modify the susceptibility to the development of steatosis and liver damage progression in patients with CHC. We and others have previously shown that the I148M variant of the *Patatin-like phospholipase domain-containing 3 (PNPLA3)* gene, the major common genetic determinant of hepatic fat content, influences the development of steatosis, and the progression towards advanced fibrosis and hepatocellular carcinoma in patients with CHC^8–11^.

Recently, the rs58542926 C > T genetic variant of the *Transmembrane 6 superfamily member 2* gene (*TM6SF2*), which encodes for the E167K aminoacidic substitution, has been identified as a determinant of hepatic fat content and lower serum lipoproteins levels^12, 13^. This mechanism is related to the inhibition of lipidation of very-low density lipoproteins (VLDLs)^14^, which cause lipid compartmentalization within hepatocytes^12, 13, 15, 16^, thereby triggering steatohepatitis and fibrogenesis^16–18^.

Subsequent studies conducted in patients with CHC suggested that the E167K variant is also associated with steatosis^19–21^ and fibrosis development^20, 21^ in this specific population. However, in the CHC setting the association with fibrosis development remains controversial^19, 22^, and the overall evidence does not support an association of the E167K variant with liver enzymes, and index of hepatic inflammation^23^.

Therefore, this study investigated the association of the *TM6SF2* E167K variant with histological liver damage (i.e., steatosis, hepatic inflammation, and fibrosis) in patients with CHC by performing a systematic review and meta-analysis of the available literature. As secondary outcomes, we also evaluated the impact of the E167K variant on circulating lipids, other metabolic traits and their interaction with TM6SF2-histological damage associations.

## Results

### Literature retrieval

**Figure 1:**
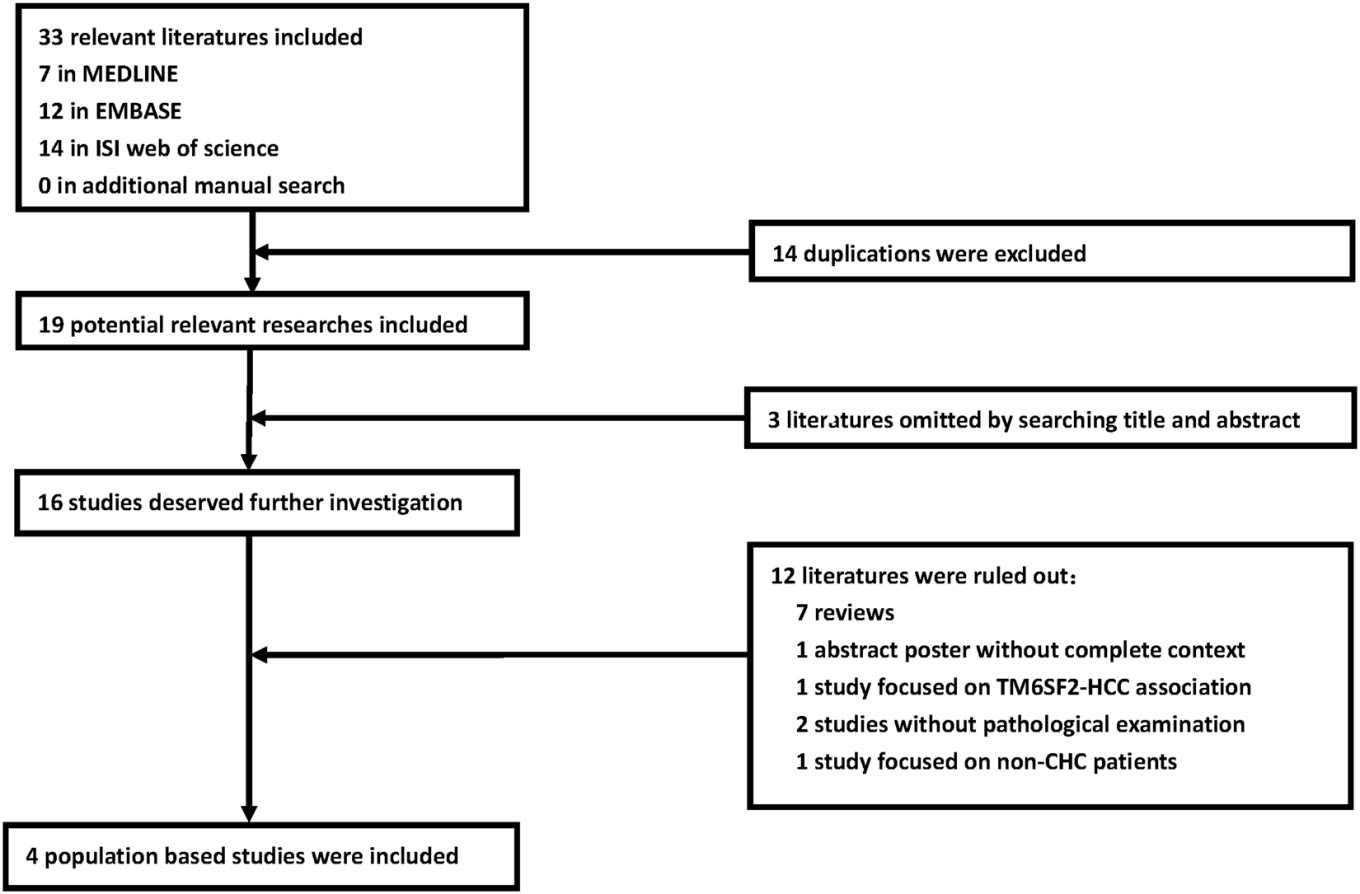
Flow chart of selected studies for meta-analysis.

The initial literature search yielded 19 potential references after excluding 14 duplicates across three databases. The manual search did not identify any additional studies. Three studies were omitted based on their titles and abstracts, and 12 studies were excluded because of specifically listed reasons. Finally, four eligible studies were selected with high inter-author consistency (Cohen’s kappa = 0.69). The flow chart is presented in Fig. 1.

### Study characteristics and quality assessment

The major characteristics of the four studies included in the analyses are presented in Table 1. Overall, 4325 patients with CHC were enrolled in the analysis. The number of enrolled patients was heterogeneous, varying from 148 to 2023 (equal to 3.4% and 46.8% of all patients, respectively). All participants were Caucasian, with a mean age that ranged between 44 and 58 years old. Nearly all of the studies selected patients naïve for anti-HCV therapy. The distribution of HCV genotypes was uneven across the studies (P < 0.05). All of the studies applied TaqMan assays for genotyping, and the genotype distribution of the *TM6SF2* E167K variant did not violate the Hardy-Weinberg equilibrium (P > 0.05), with carriage of the E167K variant (EK + KK genotypes) frequency ranging from 6.3 to 12.2%. With respect to the liver biopsy results, four studies evaluated the extent of histological steatosis/fibrosis, and three evaluated the extent of inflammation (Table 2). Histological features were classified according to different criteria^24–27^ (Table S3).

**Table 1:** Characteristics of the studies selected for meta-analysis.

First author, year, study [Reference Values for Arterial Stiffness, #2467]	Ethnicity, Country	Gender N, (women%)	Age (yr, mean ± SD)	BMI (kg/m^2^, mean ± SD)	Cohort characteristics	Study design	HCV genotype:(N)	Genotyping	*TM6SF2* E167K genotype (EE + KK%)	P-value for HWE
N. Coppola^19^	Caucasian, Italy	148,(45.2)	EE: 51 ± 12 EK + KK: 53 ± 14	EE: 26 ± 0.4 EK + KK: 24 ± 1.2	1. CHC patients; 2. no other liver disease and steatogenic medication; 3. before antiviral treatment	Hospital based cross-sectional	G1/G2/G3/G4: EE:87/28/13/2 EK + KK:14/2/2/0	TaqMan	EE/EK/KK: 130/18/0 (12.2%)	0.43
M. Milano, 2015, Milan cohort^20^	Caucasian, Italy	815,(43.6)	EE: 58 ± 13 EK + KK: 57 ± 13	EE: 24.8 ± 3.5 EK + KK: 24.5 ± 2.7	1. CHC patients; 2. no other liver disease; 3. before antiviral therapy	Hospital based cross-sectional	G1/G2/G3/G4: EE:430/202/45/69 EK + KK:35/10/20/4	TaqMan	EE/EK + KK: 746/69 (8.5%)	0.37
M. Milano, 2015, validation cohort^22^	Caucasian, Swiss/ Germany	645,(44.3)	EE: 53 ± 12 EK + KK: 50 ± 11	NA	1. CHC patients; 2. no other liver disease 3. before antiviral therapy	Hospital based cross-sectional	NA	TaqMan	EE/EK + KK: 550/95 (14.7%)	>0.05
S. Petta, 2015^22^	Caucasian, Italy	694,(45.7)	EE: 54 ± 12 EK + KK: 53 ± 12	EE: 26.6 ± 3.6 EK + KK: 25.7 ± 2.9	1. CHC patients, 2. no other liver disease	Hospital based cross-sectional	G1: 694 EE:650 EK + KK:44	TaqMan	EE/EK + KK: 650/44 (6.3%)	0.74
M. Eslam, 2016^21^	Caucasian, Australia/UK/Spain/Italy/Germany	2023,(37.7)	EE: 45 ± 11 EK: 44 ± 11 KK: 45 ± 7	EE: 26.5 ± 4.9 EK + KK: 25.8 ± 4.3	1. CHC patients; 2. no other liver disease 3. before antiviral therapy	Hospital based cross-sectional	G1/G2/G3/G4: 1335/202/445/41	TaqMan	EE/EK/KK: 1778/235/10 (12.1%)	0.55

**Table 2:** Genetic impact of *TM6SF2 E167K* polymorphism on hepatic histological features in the selected studies.

First author, publication year, study, [Reference Values for Arterial Stiffness, #2467]	Subgroup	Histological feature	Scoring system [Reference Values for Arterial Stiffness, #2467]	Number^a^: (N)	Comparison^b^	Statistics	OR(95% CI)	P-value	Adjustment
N. Coppola, 2015^19^	None	Steatosis	NAS^24^	S0/S1/S2/S3: EE:45/69/6/10 EK + KK:2/10/2/4	S2 + S3 vs. S0 + S1	Chi-square	3.56 (1.17–10.8)	0.02	None
	None	Fibrosis	Ishak^25^	F0/F1/F2/F3/F4/F5/F6: EE:3/40/35/29/14/7/2 EK + KK:2/5/3/3/3/1/1	F4-F6 vs. F0-F3	Chi-square	1.79 (0.58–5.51)	0.31	None
M. Milano, 2015, Milan cohort^20^	None	Steatosis	Ishak^25^	S0/S1/S2/S3: EE:240/383/87/36 EK + KK:15/36/11/7	S2 + S3 vs. S0 + S1	Chi-square	1.79 (1.01–3.16)	0.04	None
	Genotype3 CHC	Steatosis	Ishak^25^	NA	S(X + 1) vs. S(X)^c^	Ordinal regression	1.23 (0.60–2.52)	0.58	Age, gender, BMI, HCV G3,diabetes, alcohol intake, *PNPLA3* I148M
	Genotype (1 + 2 + 4) CHC	Steatosis	Ishak^25^	NA	S(X + 1) vs. S(X)	Ordinal regression	1.33 (1.03–1.72)	0.03	Age, gender, BMI, diabetes, alcohol intake, *PNPLA3* I148M
	None	Inflamma tion	Ishak^25^	G0-G2/G3-G4/G5-G6/ G7-G8/G9-G10/G11-G12 /G13-G18: EE:26/105/262/209/92/ 39/13 EK + KK: 0/17/26/13/5/4/4	G(X + 1) vs. G(X)^d^	Ordinal regression	1.27 (1.02–2.59)	0.04	Age, gender, BMI, HCV G3, diabetes, alcohol intake, ancestry, *PNPLA3* I148M
	None	Fibrosis	Ishak^25^	F0/F1/F2/F3/F4/F5/F6: EE:12/118/201/154/71/60/130	F6 vs. F1-F5	Logistic regression	2.19 (1.18–3.39)	0.01	Age, gender, BMI, HCV G3, diabetes, alcohol intake, ancestry, *PNPLA3* I148M
	None	Fibrosis	Ishak^25^	EK + KK: 0/9/18/10/9/2/21	F(X + 1) vs. F(X)^e^	Ordinal regression	1.23 (0.99–1.53)	0.06	Age, gender, BMI, HCV G3, diabetes, alcohol intake, ancestry, *PNPLA3* I148M
M. Milano, 2015, validation cohort^20^	None	Fibrosis	METAVIR^27^	NA	F2-F4 vs. F0-F1	Logistic regression	1.81 (1.02–3.04)	0.02	Age and gender
S. Petta, 2015^22^	None	Steatosis	NAS^24^	S0/S1/S2: EE:356/187/107 EK + KK:21/14/9	S(X + 1) vs. S(X)	Ordinal regression	1.48 (0.82–2.69)	0.19	Age, gender, BMI, HOMA-IR
	None	Inflamma tion	Scheuer^26^	G4/G0 + G1 + G2 + G3: EE: 285/365 EK + KK: 17/27	G4 vs. G0-G3	Chi-square	0.81 (0.43–1.51)	0.50	None
	None	Fibrosis	Scheuer^26^	F0-F2/F3-F4: EE:449/201 EK + KK:33/11	F3-F4 vs. F0-F2	Logistic regression	0.75 (0.34–1.63)	0.47	Age, gender, BMI, HOMA-IR, *PNPLA3* I148M, and IL-28B rs12979860
M. Eslam, 2016^21^	None	Steatosis	NAS^24^	S0/S1/S2/S3 EE:922/533/237/86 EK:100/94/22/19 KK:2/4/3/1	S2 + S3 vs. S0 + S1	Logistic regression	1.14 (1.02–1.27)	0.01	Age, gender, BMI, HOMA-IR, HCV genotype, alcohol intake, and *PNPLA3* I148M
	Genotype3 CHC	Steatosis	NAS^24^	NA	S(X + 1) vs. S(X)	Ordinal regression	1.05 (0.99–1.08)	0.50	Age, gender, BMI, HOMA-IR, and PNPLA3 I148M
	Genotype (1 + 2 + 4) CHC	Steatosis	NAS^24^	NA	S(X + 1) vs. S(X)	Ordinal regression	1.12 (1.11–1.13)	0.04	Age, gender, BMI, HOMA-IR, and *PNPLA3* I148M
	None	Inflamma tion	METAVIR^27^	G0/G1/G2/G3 EE:75/889/620/194 EK:11/129/74/21 KK:1/1/6/2	G(X + 1) vs. G(X)	Ordinal regression	1.04 (0.85–1.26)	0.10	Age, gender, steatosis, HOMA-IR, BMI, HCV genotype,alcohol intake, and *PNPLA3* I148M
	None	Fibrosis	METAVIR^27^	F0/F1/F2/F3/F4 EE:232/630/512/248 /156 EK:36/87/60/22/30 KK:1/3/0/5/1	F2-F4 vs. F0-F1	Logistic regression	1.39 (1.04–1.87)	0.02	Age, gender, steatosis, HOMA-IR, BMI, HCV genotype, alcohol intake, and *PNPLA3* I148M
					F4 vs. F0-F3	Logistic regression	1.82 (1.01–3.28)	0.01	Age, gender, steatosis, HOMA-IR, BMI, HCV genotype, alcohol intake, and *PNPLA3* I148M
					F(X + 1) vs. F(X)	Ordinal regression	1.07 (1.01–1.14)	0.04	Age, gender, steatosis, HOMA-IR, BMI, HCV genotype, alcohol intake, and *PNPLA3* I148M

^a^S, G and F respectively represent the histological severity on steatosis, inflammation and fibrosis; ^b^ *TM6SF2* E167K variant is coded in dominant genetic model (EE/EK + KK) for each comparison; ^c^S(X + 1) vs.S(X) means the continuous comparison between subgroups with adjacent advanced and mild steatosis; ^d^G(X + 1) vs.G(X) means the continuous comparison between subgroups with adjacent advanced and mild inflammation; ^e^F(X + 1) vs.F(X) means the continuous comparison between subgroups with adjacent advanced and mild fibrosis.

Regarding study quality, three of the four studies had defects in the methods and results sections, according to criteria of the STREGA statement (Table S2).

### TM6SF2 variant and steatosis severity in patients with CHC

**Figure 2:**
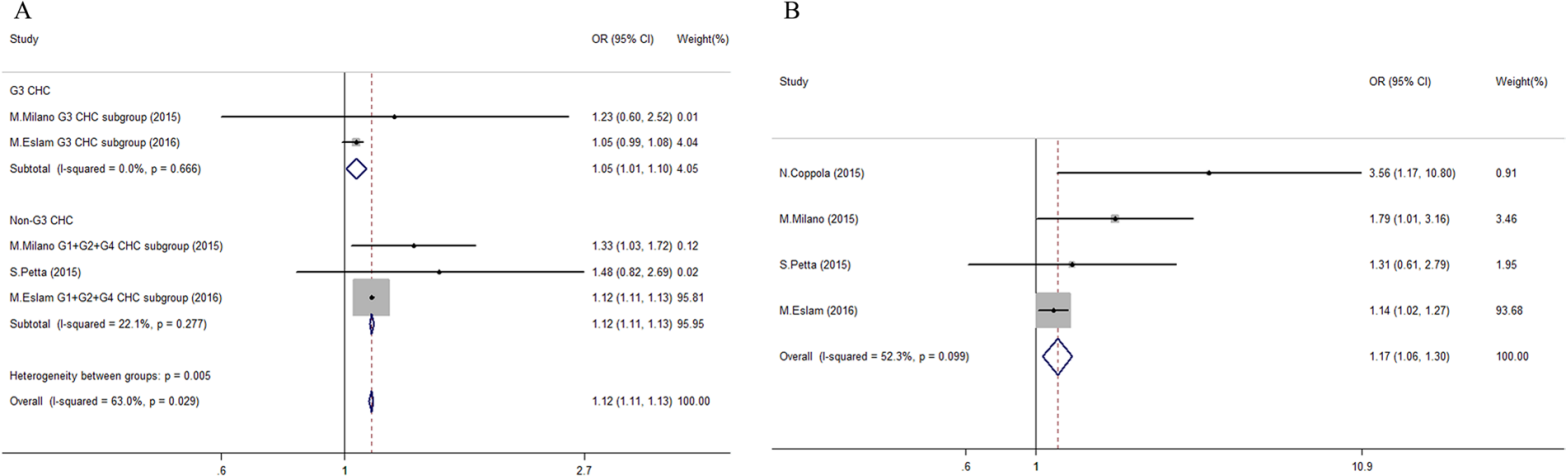
Forest plot of genetic association for *TM6SF2* E167K polymorphism on steatosis status in chronic hepatitis C patients. (**A**) Pooled continuous odds ratio of *TM6SF2* E167K variant (EK + KK) on steatosis severity in patients categorized by hepatitis C virus genotypes; (**B**) Pooled dichotomous odds ratio for *TM6SF2* E167K variant (EK + KK) on significant steatosis (≥S2) compared to non-significant steatosis group (<S2) in chronic hepatitis C patients.

The *TM6SF2* E167K variant showed a comprehensive impact on steatosis severity. Carriage of the *TM6SF2* variant had a more prominent effect on the risk of advanced steatosis in non-HCV-G3 infected patients (OR 1.12 vs. 1.05, P < 0.05, Fig. 2). Interestingly, we found that the *TM6SF2* genotype distribution was unequal in patients with CHC stratified by HCV genotype (P < 0.05). Compared with HCV-G3 patients and healthy controls, non-HCV-G3 patients had a significantly lower prevalence of E167K variant carriers (15.6% and 13.6% vs. 9.2%, respectively; P < 0.01, Figure S1).

When steatosis was treated as a dichotomous variable, it was robustly associated with the *TM6SF2* variant across the whole CHC population (pooled adjusted OR = 1.55, 95% CI = 1.23–1.94), with low inter-subgroup heterogeneity (I^2^ = 0%, P = 0.55, Figure S2A). An increased prevalence of severe steatosis (≥S3) was also observed in carriers of the E167K variant (12.6% vs. 7.2%, respectively; pooled adjusted OR = 2.19, 95% CI = 1.48–3.24; Figure S2B).

An additional subgroup analysis revealed that certain demographic characteristics such as younger age, male gender, higher BMI and a larger sample size of the study were associated with a reduced impact of the *TM6SF2* variant on steatosis severity (P < 0.05, Table 3 left panel), which is consistent with the results of the sensitivity analysis (Figure S3A). A cumulative meta-analysis also found decreased but stable effects of the *TM6SF2* variant on steatosis as the sample sizes increased (Figure S4A).

**Table 3:** Subgroup analysis on genetic association among *TM6SF2* E167K polymorphism and histological features in chronic hepatitis C patients.

Group	Steatosis				Fibrosis			
	Data points (n)	Pooled OR^a^ (95% CI)	P1^c^	P2^d^	Data points (n)	Pooled OR^b^ (95% CI)	P1	P2
Mean age(yr)								
≤50	1	1.14 (1.02–1.27)	NA		1	1.39 (1.04–1.87)	NA	
>50	3	1.80 (1.18–2.74)	0.347	0.041	4	1.29 (0.94–1.78)	0.339	0.745
Gender								
Women% <40%	1	1.14 (1.02–1.27)	NA		1	1.39 (1.04–1.86)	NA	
Women% >40%	3	1.80 (1.18–2.74)	0.347	0.041	4	1.29 (0.94–1.78)	0.339	0.745
Mean BMI(kg/m^2^)								
≤26	2	2.07 (1.24-3.43)	0.281		2	1.23 (0.79–1.93)	0.953	
>26	2	1.14 (1.03–1.27)	0.723	0.025	2	1.29 (0.98–1.70)	0.148	0.869
Sample size(n)								
≤1000	3	1.80 (1.18-2.74)	0.347		4	1.29 (0.94–1.78)	0.339	
>1000	1	1.14 (1.02–1.27)	NA	0.041	1	1.39 (1.04–1.86)	NA	0.745
Naïve for antiviral therapy								
Yes	3	1.17 (1.05–1.30)	0.045		4	1.41 (1.13–1.77)	0.763	
No	1	1.31 (0.61–2.80)	NA	0.774	1	0.75 (0.34–1.64)	NA	0.129
Prevalence of G3 CHC(%)								
≤10%	2	1.60 (1.01–2.52)	0.520		2	1.07 (0.70–1.64)	0.290	
>10%	2	1.15 (1.03–1.29)	0.046	0.171	2	1.37 (1.04–1.82)	0.782	0.337
Prevalence of EK + KK carriers(%)								
≤10%	2	1.60 (1.01-2.52)	0.520		2	1.07 (0.70–1.64)	0.290	
>10%	2	1.15 (1.03–1.29)	0.046	0.171	3	1.46 (1.13–1.87)	0.654	0.222
Adjustment of *PNPLA3* I148M								
No	2	1.80 (0.96–3.37)	0.146		3	1.44 (1.02–2.04)	0.567	
Yes	2	1.16 (1.04–1.29)	0.128	0.174	2	1.29 (0.98–1.70)	0.148	0.623
Adjustment of steatosis presence								
No					4	1.29 (0.94–1.78)	0.339	
Yes					1	1.39 (1.04–1.87)	NA	0.745
Scoring system^e^								
Ishak^25^					2	1.23 (0.79–1.93)	0.953	
METAVIR^27^					2	1.47 (1.14–1.91)	0.404	
Scheuer^26^					1	0.75 (0.34–1.64)	NA	0.251

^a^OR represents the risk of significant steatosis for EK + KK carriage compared to EE group; ^b^OR represents the risk of significant fibrosis for EK + KK carriage compared to EE group; ^c^P1 represents the heterogeneity of pooled result within each subgroup; ^d^P2 represents the inter-subgroup heterogeneity across studies categorized by the same criteria; ^e^Comparison was only applied for fibrosis because no difference observed in criteria defining the steatosis severity.

### TM6SF2 variant and inflammation activity in patients with CHC

**Figure 3:**
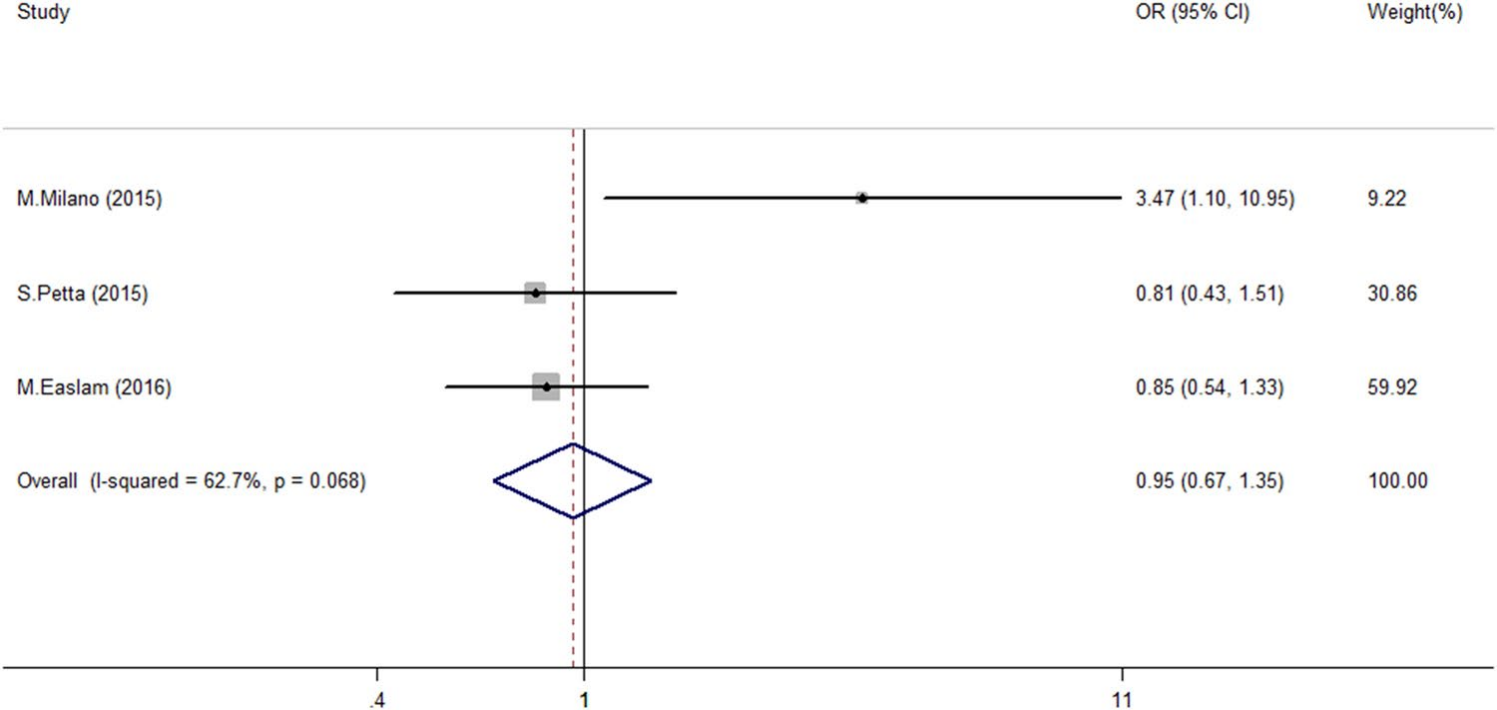
Forest plot of genetic association for *TM6SF2* E167K polymorphism on inflammation status in chronic hepatitis C patients. Pooled dichotomous odds ratio of *TM6SF2* E167K variant (EK + KK) on severe inflammation^a^ compared to non-severe inflammation group in chronic hepatitis C patients. ^a^Severe inflammation was defined as G13-G18 status in study applying Ishak criteria^25^ and G4 status in study applying Scheuer^26^ and METAVIR^27^ criteria.

Less information was available in the literature regarding the association between *TM6SF2* variation and hepatic inflammation (Table 2). For continuous comparisons, the pooled risk on inflammation progression was 1.14, but the 95% CI ranged across 1 (Figure S5A). Regarding the dichotomous comparison, the *TM6SF2* variant was not associated with more severe inflammatory activity (Fig. 3 and Figure S5B). Importantly, overall study results were uncertain with significant inconsistency (I^2^ ranged from 42.6 to 79.7%). Only Milano M. *et al*., detected a positive association between the *TM6SF2* E167K variant and severe inflammation. However, a sensitivity analysis did not reveal any specific study as a potential confounder that influenced the overall results (Figure S3B). These data suggest that the *TM6SF2* variant had no to little effect on the development of hepatic inflammation in patients with CHC.

### TM6SF2 variant and fibrosis in patients with CHC

**Figure 4:**
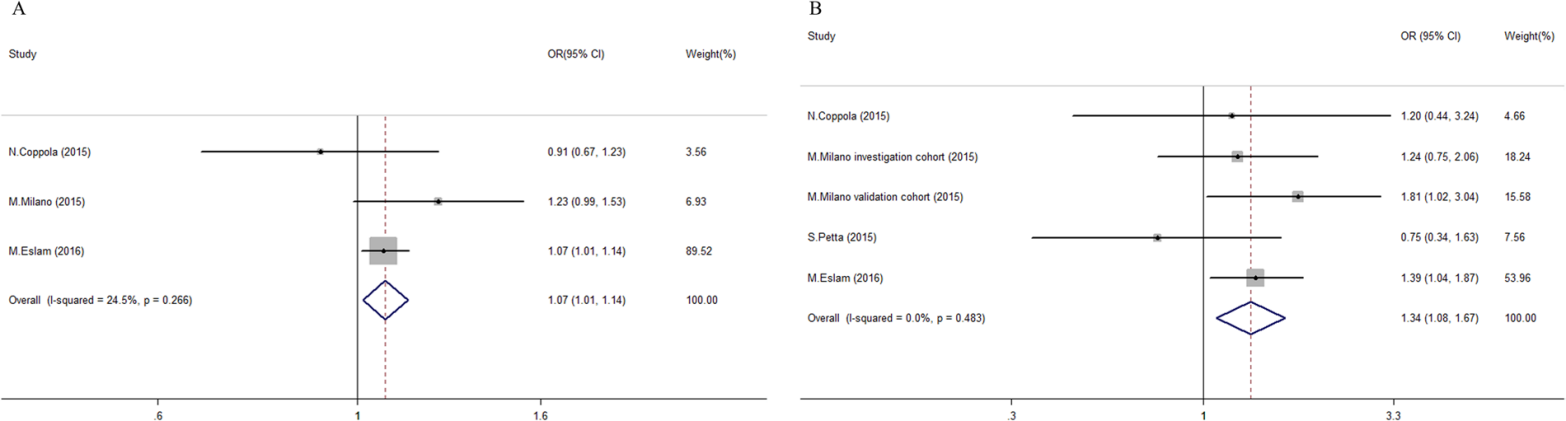
Forest plot of genetic association for *TM6SF2* E167K polymorphism on fibrosis status in chronic hepatitis C patients. (**A**) Pooled continuous odds ratio of *TM6SF2* E167K variant (EK + KK) on advanced fibrosis severity in chronic hepatitis C patients; (**B**) Pooled dichotomous odds ratio for *TM6SF2* E167K variant (EK + KK) on significant fibrosis^a^ compared to non-significant fibrosis group in chronic hepatitis C patients. ^a^Significant fibrosis was defined as ≥ F3 in studies applying for Ishak criteria^25^, and ≥ F2 in studies applying for METAVIR criteria^27^.

The *TM6SF2* variant showed a robust association with fibrosis development in patients with CHC. For continuous comparisons, the presence of E167K increased the risk of advanced fibrosis by 7% (95% CI = 1.01–1.14, Fig. 4A), with low heterogeneity (I^2^ = 24.5%, P = 0.266; Fig. 4A). Regarding the dichotomous comparisons, the *TM6SF2* variant was also associated with significant fibrosis (pooled OR = 1.34, 95% CI = 1.08–1.67) without obvious discrepancy (I^2^ = 0%, P = 0.779), despite the classification using diverse scoring systems (Fig. 4B). Importantly, the prevalence of cirrhosis was higher among E167K carriers than non-carriers (16.0 vs. 10.8%, respectively). Indeed, after adjusting for potential covariates (Table 2), the *TM6SF2* variant was significantly associated with an approximately two-fold higher risk of cirrhosis (pooled OR = 2.05, 95% CI = 1.39–3.02; Figure S6). In contrast to what was observed for steatosis, a subgroup analysis did not demonstrate any confounds with obvious interference on the *TM6SF2*-fibrosis association (all *P* > 0.05, Table 3 right panel). A sensitivity analysis did not find any specific study that significantly influenced the pooled OR of significant fibrosis (Figure S3C). The results of the cumulative meta-analysis found that the genetic effects of the *TM6SF2* variant on fibrosis severity became significant and more stable after the results from the larger sample of Eslam *et al*. were included (Figure S4B).

### TM6SF2 variant and circulating lipids in patients with CHC

**Figure 5:**
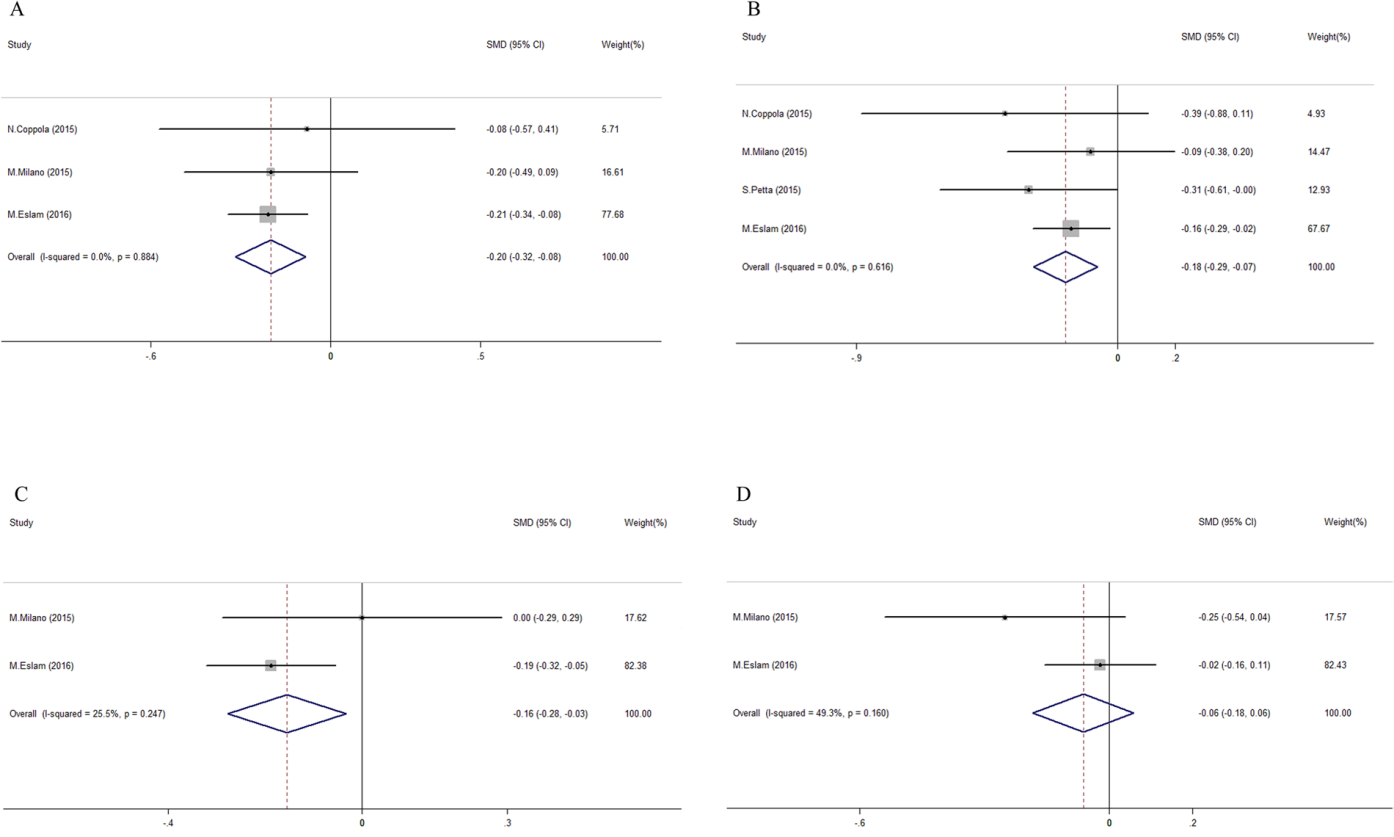
Forest plot for genetic impact of *TM6SF2* E167K polymorphism on circulating lipids in chronic hepatitis C patients. (**A**) Pooled standardized mean differences of triglyceride level in subgroups with different *TM6SF2* E167K polymorphism (EK + KK vs. EE); (**B**) Pooled standardized mean differences of total cholesterol level in subgroups with different *TM6SF2* E167K polymorphism (EK + KK vs. EE); (**C**) Pooled standardized mean differences of low density lipoprotein cholesterol level in subgroups with different *TM6SF2* E167K polymorphism (EK + KK vs. EE); (**D**) Pooled standardized mean differences of high density lipoprotein cholesterol level in subgroups with different *TM6SF2* E167K polymorphism (EK + KK vs. EE).

All of the considered studies reported the effect of the *TM6SF2* E167K variant on circulating lipids. The E167K variant was associated with decreased levels of circulating lipids in patients with CHC (Fig. 5). Quantitative comparisons revealed that E167K carriers had approximately 12.0% lower TG levels (1.03 ± 0.52 vs. 1.17 ± 0.67 mmol/L; pooled SMD = 0.20, 95% CI = 0.08–0.32 mmol/L, I^2^ = 0%, Fig. 5A), and 6.1% lower TC levels (4.28 ± 0.93 vs. 4.56 ± 1.52 mmol/L, pooled SMD = 0.18, 95% CI = 0.07–0.29 mmol/L, I^2^ = 0%; Fig. 5B) than non-carriers. LDL-C but not HDL-C was lower in carriers of the E167K variant (pooled SMD = 0.16, 95% CI = 0.03–0.28 mmol/L). However, this latter analysis was based on only two data points with moderate heterogeneity (I^2^ = 25.5% and 49.3% for LDL-C and HDL-C in Fig. 5C and D, respectively).

### Association of TM6SF2 variant with metabolic traits and viral load in patients with CHC

The impact of the *TM6SF2* variant on glucose levels and insulin resistance were reported in three and two studies, respectively (Figure S7). Convincing evidence showed negative results for the comparison between carriers with the *TM6SF2* variant on fasting glucose value (5.23 ± 0.81 vs. 5.30 ± 1.47 mmol/L, pooled SMD = −0.05, 95% CI = −0.17/0.07 mmol/L) and HOMA-IR (2.48 ± 3.26 vs. 2.45 ± 4.66, pooled SMD = 0.02, 95% CI = −0.10/0.15, both I^2^ = 0%).

The *TM6SF2* variant was associated with lower BMI (i.e., carriers of the E167K mutation tended to be leaner; Figure S8), but with high heterogeneity (I^2^ = 97.2%). However, after excluding the study by Coppola *et al*., which had a smaller sample size and inferior study quality, carriage of the E167K variant was associated with a 2% decrease in BMI (25.6 ± 3.9 vs. 26.1 ± 4.4 kg/m^2^; pooled SMD = 0.15, 95% CI = 0.04–0.26 kg/m^2^; data not shown) in the absence of obvious heterogeneity (I^2^ = 0%).

All four studies compared the HCV-RNA load in subgroups stratified by the *TM6SF2* genotype (Figure S9). HCV-RNA levels did not differ in carriers with the E167K variant vs. non-carriers (5.70 ± 1.62 vs. 5.77 ± 1.57; quantitative HCV RNA after log transformation). However, a moderate to high inconsistency was observed (I^2^ = 58.9%).

### Phenotype-disease association based on the Mendelian randomization analysis

TGs and TC were selected as candidate phenotypes for the Mendelian randomization (MR) analysis of the for stable association with *TM6SF2* variant in high consistence (I^2^ < 25%). As Figure S10 shows, we found approximately 52% (OR = 0.48, 95% CI: 0.33–0.68) and 58% (OR = 0.42, 95% CI: 0.29–0.65) decreases in the risk of more severe steatosis per 1 mmol/L increase in circulating TG and TC levels, respectively. However, the causal effect of TG or TC variation was nonsignificant in patients with advanced fibrosis stages (the 95% CI of OR_phenotype-disease_ ranged across 1; Figure S10).

### Publication bias analysis

Begg’s funnel plot was applied to examine publication bias (Figure S11). Although a slight asymmetry was observed in this plot (Figure S11A), no significant publication bias was detected using either Egger’s or Begg’s tests (P > 0.05).

## Discussion

The current study performed a systematic review and meta-analysis of the literature concerning the effect of the *TM6SF2* E167K variant on liver disease severity in patients with CHC, while also addressing its influence on metabolic and viral features. The major finding are that the *TM6SF2* E167K variant favors the development of steatosis and fibrosis, but not of inflammation, in individuals with CHC infection. In our analyses, E167K was associated more robustly with fibrosis than with steatosis. This result might be related to known higher inter-observer variability in the classification of steatosis than fibrosis that is consistently reported across studies^28^, as well as to the well-established steatogenic effect of infection via HCV-G3^29^. Alternatively, the measurement of hepatic intracellular lipid content via histology might underestimate the disturbance in lipid handling among patients with advanced liver fibrosis because of the reduction in hepatic lipid accumulation with progressive accumulation of extracellular matrix in the liver^30^.

Importantly, carriage of the *TM6SF2* E167K variant conferred a stable and significant predisposition towards the development of progressive hepatic fibrosis. In particular, our pooled results found that the *TM6SF2* variant predicted the risk of cirrhosis. Overall, the results of the present analysis suggest that carriage of the E167K *TM6SF2* variant predicts an increased risk of clinically significant and advanced fibrosis in patients with CHC. The same conclusion might be drawn for the presence of steatosis in general because histological steatosis is associated with faster fibrosis progression independent of its aetiology^31^, and other genetic determinants of hepatic fat content, (e.g., the *PNPLA3* I148M and *MBOAT7* variation) are associated with fibrosis development patients with CHC^8, 10, 32^. Because the impact of E167K *TM6SF2* on fibrosis progression is not restricted to patients with CHC^16, 17, 33^, and carriers of the variant have more advanced fibrosis at diagnosis on average, future studies should determine whether this genetic factor can be used to predict the risk of liver-related complications after viral eradication.

Furthermore, the *TM6SF2* variant was associated with lower circulating serum lipids, primarily on TGs and non-HDL cholesterol. This finding is consistent with the model in which the *TM6SF2* variant favours steatosis development because of decreased VLDL secretion and lipidation^12–14, 34, 35^. Using an MR analysis as an integrated approach across genotypes, phenotypes, and histological features, we found that the genetic susceptibility conferred by the *TM6SF2* variant on steatosis (but not fibrosis) severity was influenced by lipid phenotypes with regard to their causal effects on lipid accumulation in patients with CHC. In fact, 1 unit (mmol/L) of decrease in TG/TC level might increase the susceptibility of steatosis approximately 2-folds. Importantly, the *TM6SF2* variation showed a common but distinguished impact on steatosis both in patients with G3 and those with non-G3 HCV infection, suggesting that the *TM6SF2* variant independently influences hepatic fat accumulation even in the presence of the strong viral factors that inhibit VLDL secretion via other mechanisms^6, 36, 37^. Therefore, the dissociation of the E167K variant between more severe liver damage but more favourable lipid profile observed in the general population and non-alcoholic fatty liver disease (NAFLD) patients is also present in patients with CHC.

The association between the *TM6SF2* genotype and liver damage was not confounded by metabolic cofactors. Indeed, in keeping with observations of the general population^12, 22^ and patients with NAFLD^16, 34^, the E167K variant was not associated with an increased risk of hyperglycaemia or insulin resistance. Intriguingly, the current summary of the available evidence revealed an association between carriage of the *TM6SF2* E167K variant and slightly reduced BMI. Although it is tempting to speculate that the association might depend on impairments in chylomicrons and VLDL secretion (thereby supporting adipose tissue growth in carriers of the mutation), we must remember that the E167K variant does not affect BMI in the general population^12^. Conversely, the E167K variant was associated with an increased risk of cirrhosis, an important cause of malnutrition and low BMI, which might have driven the observed association^38^. Additional epidemiological investigations evaluating single patient data are required to confirm this association.

One important difference between the results of the present analysis and findings derived from studies conducted in patients with NAFLD^16^ is that the *TM6SF2* variation was not associated with histological hepatic inflammation in CHC. This is possibly due to interference of the E167K variant in the process of VLDL secretion that impairs HCV viremia and infectivity^39^. This hypothesis is also supported by the evidence collected in genetic studies of individuals with NAFLD, in whom genetic susceptibility to hepatic lipid accumulation is the major determinant of liver damage progression^40, 41^. One important caveat to note is that steatosis and *TM6SF2* variation increase the risk of lobular necroinflammation with the infiltration of granulocytes^34^, a typical feature of non-alcoholic steatohepatitis^24^. Conversely, histological scores developed for CHC primarily capture the portal and peri-portal infiltration of lymphocytes, and can miss the nonalcoholic steatohepatitis (NASH)-related features of liver damage possibly associated with *TM6SF2* variation. Nevertheless, our findings reinforce the notion that circulating aminotransferase levels, which are not affected by *TM6SF2* variation in patients with CHC^23^, are not an accurate predictor of the severity of damage and fibrosis.

The limitations of our findings include the following. First, the main results were pooled from only four papers based on participants of European-descent selected using strict criteria; however, the completion of a meta-analysis on the impact of the TM6SF2 variant on hepatic damage and the assessment of potential controversies across studies containing patients with CHC is noteworthy. Second, attention should be focused on the lack of stability of the pooled results for different scoring systems applied across studies (Table S3). Third, we chose aggregate data (AD) in the enrolled literature with clinical and statistical inferiority to individual participant data (IPD). In our case, however, the OR was calculated based on the raw data provided in original tables and histograms from the literature, which are considered equivalent to an IPD meta-analysis^42^. Nevertheless, IPD meta-analyses of studies, are still needed to reach even more reliable conclusions less subjected to potential bias. Fourth, a lack of adjustment for steatogenic factors such as alcohol intake and less consistent data clusters in the subgroup analysis might have biased the pooled results. Finally, despite the uneven distribution of *TM6SF2* genotypes across patients infected by diverse HCV genotypes (Figure S1), we were unable to evaluate the impact of *TM6SF2* variation on fibrosis development in subgroups stratified by HCV genotypes because of the lack of available information.

In conclusion, by systematically reviewing and meta-analysing the available literature, we found that the *TM6SF2* E167K variant is associated with an increased predisposition towards the development of the full spectrum of steatosis and fibrosis (but not inflammation) in individuals of European descent with CHC infection. This variant is associated with a reduction in circulating non-HDL cholesterol and TG levels. Decreased TC and TG levels might amplify the genetic susceptibility of TM6SF2 variant on advanced steatosis.

## Experimental Procedures

### Literature retrieval and selection

A comprehensive search for literature addressing the genetic associations of *TM6SF2* variants on hepatic steatosis and relevant complications in patients with CHC was conducted using the Medline, EMBASE, and the Cochrane Library databases without language restriction (updated until May 20, 2017). Additionally, we manually searched for “grey” literature on website (e.g., the BIOSIS, EAGLE, and INIST databases) according to the guidance of the Cochrane Handbook for Systematic Reviews (Version 5.1.0, http://handbook.cochrane.org/front_page.htm). The reference lists of enrolled studies and publications with citations of included papers were also reviewed for suitable papers. Our literature search strategy is shown in Table S1. Relevant papers were initially selected after browsing title and abstract, and then two authors (ZT L and SP Q) reviewed the full text.

Qualified studies were selected when they met the following criteria: 1. Patients were clearly diagnosed with CHC but were free of other severe liver/systemic disease; 2. The *TM6SF2* E167K variant was genotyped using reliable methods; 3. Histological features were assessed via liver biopsy under the guidance of a pre-defined scoring system; 4. The risk of hazardous variants on the susceptibility of hepatic histological lesions was reported or could be calculated. If two (or more) studies included the same cohort, then the most recent study was included.

### Data extraction

The following information provided by the enrolled studies was extracted: study name, population characteristics (e.g., ethnicity, gender, age, body mass index [BMI)), study design, HCV genotype distribution, and *TM6SF2* genotyping (methods, number of carriers, and Hardy-Weinberg equilibrium *P* value). Then, additional information concerning the association between the *TM6SF2* E167K polymorphism and specific histological features (categorised by steatosis, inflammation and fibrosis) including the scoring system, number of patients with different disease severities classified by *TM6SF2* variants, comparisons, corresponding odds ratios (ORs), and adjusted covariates were also collected. In addition, quantitative indicators of lipids (triglycerides [TGs], total cholesterol [TC), low density lipoprotein cholesterol [LDL-C), high density lipoprotein cholesterol [HDL-C)), insulin resistance (fasting blood glucose, homeostatic model assessment insulin resistance index [HOMA-IR^43^]), and viral load of HCV RNA, categorised by *TM6SF2* variants, were collected when available. The corresponding authors of the original studies were contacted via e-mail for data missing from the published papers when necessary.

### Quality assessment

The qualities of the enrolled studies were systematically assessed using the pre-recommended criteria from Strengthening the Reporting of Genetic Association (STREGA) study guidelines^44^. Eleven items were included in the qualitative checklist (Table S2).

### Statistical analysis

We adopted a dominant model (EK + KK *vs*. EE) to evaluate the genetic impacts, because of low prevalence of carriers with the homozygous variant (KK). Quantitative phenotypes that used different units across studies were unified before comparisons. For covariates reported in participants with CT and TT genotypes, the data were combined according to the number (N), mean and standard deviation (SD) using pre-defined formulas^45^.

If the original results were not reported, then data were extracted using Engauge Digitizer version 4.1 (http://digitizer.sourceforge.net/). Reported medians and interquartile ranges were transformed to mean values and SDs according to pre-defined methods^46^. The OR (if not given) and deviation of genotype distribution taken from the Hardy-Weinberg equilibrium were tested using the chi-square test.

The difference in quantitative phenotypes was evaluated via the pooled standard mean difference (SMD) and corresponding 95% confidence intervals (CIs). With respect to the categorical variables, pooled ORs and 95% CIs were used to assess the genetic effects on disease predisposition. Pooled ORs and SMDs were calculated using Metan^47^. The continuous risks of *TM6SF2* variation on the severity of the histological features were evaluated via the generalised least squares (GLST) method^48^. We separately calculated the pooled risk stratified by potential confounds to assess their impacts on the associations between the TM6SF2 variant and histological features. A cumulative meta-analysis was performed to identify the change in effect as the study sample sizes increased. A sensitivity analysis was performed by omitting one study sequentially to examine its effect on the overall results^49^. Publication bias was evaluated using Egger’s funnel plot and Begg’s test^50^.

The causal effects of the quantitative phenotypes on the genetic associations between the *TM6SF2* E167K polymorphism and histological severity (primarily steatosis and fibrosis) were examined via a Mendelian randomisation (MR) approach in absence of reverse causation and unadjusted confounds^51, 52^. In our study, exposure indicators, which were more strongly correlated with the TM6SF2 variant in low heterogeneity (P < 0.05, I^2^ < 25%) were selected for the MR analysis. The risk associated with carrying the TM6SF2 variant on more severe steatosis/fibrosis was defined as OR_EK/KK vs. EE_, and the mean difference on the quantitative phenotype was defined asΔP. OR_phenotype-disease_ was calculated using OR_EK/KK vs. EE_^1/ΔP^ as a non-confounded effect with confounders on the risk of more severe steatosis/fibrosis per 1 unit of elevation on the candidate phenotype.

The heterogeneity caused by discrepancies across different studies was assessed using the I^2^ test. Thresholds of 25%, 50%, and 75% were considered as representing low, moderate, and high heterogeneities, respectively^53^. A fixed-effect model was used in the case of nonsignificant heterogeneity (*P* > 0.05, I^2^ < 50%), and a random-effects model was used for other conditions. All analysis was performed by STATA 12.0 (College station, TX, USA). *P* < 0.05 was considered significant.

## Electronic supplementary material

Supplementary files
