# Supplementary material for: The effect of the TM6SF2 E167K variant on liver steatosis and fibrosis in patients with chronic hepatitis C: a meta-analysis
Source: Sci Rep. 2017 Aug 24;7:9273. doi: 10.1038/s41598-017-09548-9 (PMC5571206; doi:10.1038/s41598-017-09548-9)

**The effect of the *TM6SF2* E167K variant on liver steatosis and fibrosis in patients with chronic hepatitis C : a meta-analysis**

Zhengtao Liu^1,2a^, Shuping Que^3a^, Lin Zhou^2^, Shusen Zheng^2^, Stefano Romeo^4,5^,

Adil Mardinoglu^1,6^,* & Luca Valenti^7,8^,*

1. Science for Life Laboratory, KTH - Royal Institute of Technology, SE-171 21, Stockholm, Sweden

2. Key Laboratory of Combined Multi-Organ Transplantation, Ministry of Public Health, First Affiliated Hospital, School of Medicine, Zhejiang University, Hangzhou, 310003, China

3. Department of Pediatrics, Women and children's hospital of Guangxi, Nanning, 530005, Guangxi province, China

4. Sahlgrenska Center for Cardiovascular and Metabolic Research, Wallenberg Laboratory, Department of Molecular and Clinical Medicine, Department of Cardiology, University of Gothenburg, SE-413 45, Sweden

5. Clinical Nutrition Unit, Department of Medical and Surgical Sciences, University Magna Graecia, Catanzaro, 88100, Italy

6. Department of Biology and Biological Engineering, Chalmers University of Technology, Gothenburg, Sweden

7. Department of Pathophysiology and Transplantation, Università degli Studi di Milano, Italy

8. Internal Medicine, Fondazione IRCCS Ca’ Granda Ospedale Policlinico Milano, Italy

*Corresponding authors

E-mail: [adilm@scilifelab.se](mailto:adilm@scilifelab.se) (Adil Mardinoglu), [valenti@unimi.it](mailto:valenti@unimi.it) (Luca Valenti)

^a^ these authors contributed equally to the work.

**SUPPLEMENTARY TABLES**

**Table S1.** Search strategy for literatures about the association between TM6SF2 E167K variant and steatosis

| MEDLINE | Search strings | Results |
| --- | --- | --- |
| 1 | TM6SF2 [ALL Text] | 93 |
| 2 | Transmembrane 6 Superfamily Member 2 [All Text] | 39 |
| 3 | rs58542926 [All Text] | 29 |
| 4  5  6  7  8 | 1 OR 2 OR 3  Steatosis  Inflammation  Fibrosis  5 OR 6 OR 7 | 100  15497  438031  176812  600316 |
| 9 | HCV [All Text] | 50701 |
| 10 | hepatitis C virus [All Text] | 48006 |
| 11 | chronic hepatitis C [All Text] | 17959 |
| 12 | CHC [Text Word] | 4133 |
| 13 | 9 OR 10 OR 11 OR 12 | 69937 |
| 14 | 4 AND 8 AND 13 | 7 |
| Embase |  |  |
| 1 | 'TM6SF2'/exp | 124 |
| 2 | 'TM6SF2'/exp OR 'Transmembrane 6 Superfamily Member 2' | 133 |
| 3 | 'TM6SF2'/exp OR 'rs58542926' | 125 |
| 4  5  6  7  8 | 1 OR 2 OR 3  'steatosis'/exp OR steatosis  'inflammation'/exp OR inflammation  'fibrosis'/exp OR fibrosis  5 OR 6 OR 7 | 133  26122  3291501  341633  3517949 |
| 9 | 'HCV'/exp OR 'hepatitis C virus' | 100315 |
| 10 | 'CHC'/exp OR 'chronic hepatitis C' | 32335 |
| 11 | 9 OR 10 | 111808 |
| 12 | 4 AND 8 AND 11 | 12 |
| ISI |  |  |
| 1 | Topic=('TM6SF2') | 88 |
| 2 | Topic=('Transmembrane 6 Superfamily Member 2') | 162 |
| 3 | Topic=('rs58542926') | 23 |
| 4  5  6  7  8 | 1 OR 2 OR 3  Topic=('steatosis')  Topic=('inflammation')  Topic=('fibrosis')  5 OR 6 OR 7 | 225  19362  414691  177124  578732 |
| 9 | Topic=('HCV') | 58505 |
| 10 | Topic=('hepatitis C virus') | 86296 |
| 11 | Topic=('CHC') | 5094 |
| 12 | Topic=('chronic hepatitis C') | 49661 |
| 13 | 9 OR 10 OR 11 OR 12 | 122,320 |
| 10 | 4 AND 8 AND 13 | 14 |

**Table S2.** Quality assessment for genetic association studies enrolled in meta-analysis

| Study | Introduction | Method | | | | | Results | | | | |
| --- | --- | --- | --- | --- | --- | --- | --- | --- | --- | --- | --- |
|  | State objectives and  hypothesis (N/Y) | Information on the criteria for selection of participants is clearly given (N/Y) | Consideration  of the Hardy Weinberg Equilibrium in enrolled cohort (N/Y) | State replicative effort of genetic association study (N/Y) | Describe laboratory methods for genotyping (N/Y) | State the used software version and chosed options (N/Y) | State the number of subjects with different genotypes (N/Y) | Conformed to the Hardy Weinberg Equilibrium in enrolled cohort  (N/Y) | Report phenotypes in each genotype category over time (N/Y) | Report variables in subgroups categorized by potential confounder (like HCV genotype)  (N/Y) | Report genetic risks adjusted by potential confounders (age, gender, BMI et al) (N/Y) |
| N.Coppola et al, 2015  [19] | Y | Y | N | N | Y | Y | Y | Y | Y | N | N |
| M.Milano et al, 2015 [20] | Y | Y | Y | Y | Y | Y | Y | Y | Y | Y | Y |
| S.Petta  et al, 2015  [22] | Y | Y | Y | N | Y | Y | Y | Y | Y | N | Y |
| M.Eslam  et al, 2016  [21] | Y | Y | Y | N | Y | Y | Y | Y | Y | Y | Y |

**Table S3.** Criteria of scoring system applied in selected studies

|  | Steatosis | | Inflammation | | Fibrosis | |
| --- | --- | --- | --- | --- | --- | --- |
| Criteria  (name, reference) | Microscopic presentation | Score | Microscopic presentation | Score | Microscopic presentation | Score |
| NAS[24] | Grade: |  | NR | NR | NR | NR |
|  | Fat content <5%/5%-35%/35%-66%/>66% | 0/1/2/3 |  |  |  |  |
|  | Distribution: |  |  |  |  |  |
|  | Zone3/zone1/azonal/  panacinar | 0/1/2/3 |  |  |  |  |
|  | Contiguous patches |  |  |  |  |  |
|  | Not present/present | 0/1 |  |  |  |  |
|  |  |  |  |  |  |  |
| Ishak  [25] | Equal to NAS[24] | Equal to NAS[24] | Piecemeal necrosis: |  | None | 0 |
|  |  |  | Absent/in few portal area/in most portal area/continuous around< 50% of tracts/ continuous around>50% of tracts | 0/1/2/3/4 | Fibrous expansion of some portal areas | 1 |
|  |  |  | Confluent necrosis: |  | Fibrous expansion of most portal areas | 2 |
|  |  |  | Absent/focal/zone 3 in some area/zone 3 in most area/zone3+occasional P-C bridging/zone3+ multiple P-C bridging/ panacinar or multiacinar necrosis | 0/1/2/3/4/5/6 | Fibrous expansion of most portal areas with occasional P-P bridging | 3 |
|  |  |  | Focal lytic necrosis, apoptosis and focal inflammation |  | Fibrous expansion of portal areas with marked bridging (P-P) as well as P-C | 4 |
|  |  |  | Absent/<1/2-4/5-10/>10 foci per 100 field | 0/1/2/3/4 | Marked bridging (P-P and/or P-C) with occasional nodules | 5 |
|  |  |  | Portal inflammation |  | Definite cirrhosis | 6 |
|  |  |  | None/mild/moderate/moderate-marked/marked | 0/1/2/3/4 |  |  |
|  |  |  |  |  |  |  |
| Scheuer  [26] | NR | NR | Portal/periportal activity |  | None | 0 |
|  |  |  | None/portal inflammation/mild piecemeal necrosis/moderate piecemeal necrosis/severe piecemeal necrosis | 0/1/2/3/4 | Enlarged, fibrotic portal tracts | 1 |
|  |  |  | Lobular activity |  | Periportal or P-P septa | 2 |
|  |  |  | None/inflammation/focal necrosis/severe focal cell damage/damage includes bridging necrosis | 0/1/2/3/4 | Fibrosis with architectural distortion | 3 |
|  |  |  |  |  | Definite cirrhosis | 4 |
| METAVIR  [27] | NR | NR | Piecemeal necrosis |  | No fibrosis | 0 |
|  |  |  | Absent/focal periportal plate alteration in some portal tracts/diffuse periportal plate alteration in some portal tracts/diffuse periportal plate alteration in all portal tracts | 0/1/2/3 | Portal fibrosis without septa | 1 |
|  |  |  | Lobular necrosis |  | Portal fibrosis with rare septa | 2 |
|  |  |  | Less than one necroinflammatory foci per lobule/at least one necroinflammatory foci per lobule/  several necroinflammatory foci per lobule | 0/1/2 | Numerous septa without cirrhosis  Cirrhosis | 3  4 |

NR means not referred in selected studies

**SUPPLEMENTARY FIGURES**

**Figure S1.** Prevalence of the *TM6SF2* E167K variants in subjects with different hepatitis C virus genotypes

Inter-subgroup comparisons on percentage of EK+KK carriers were performed by chi-square test.


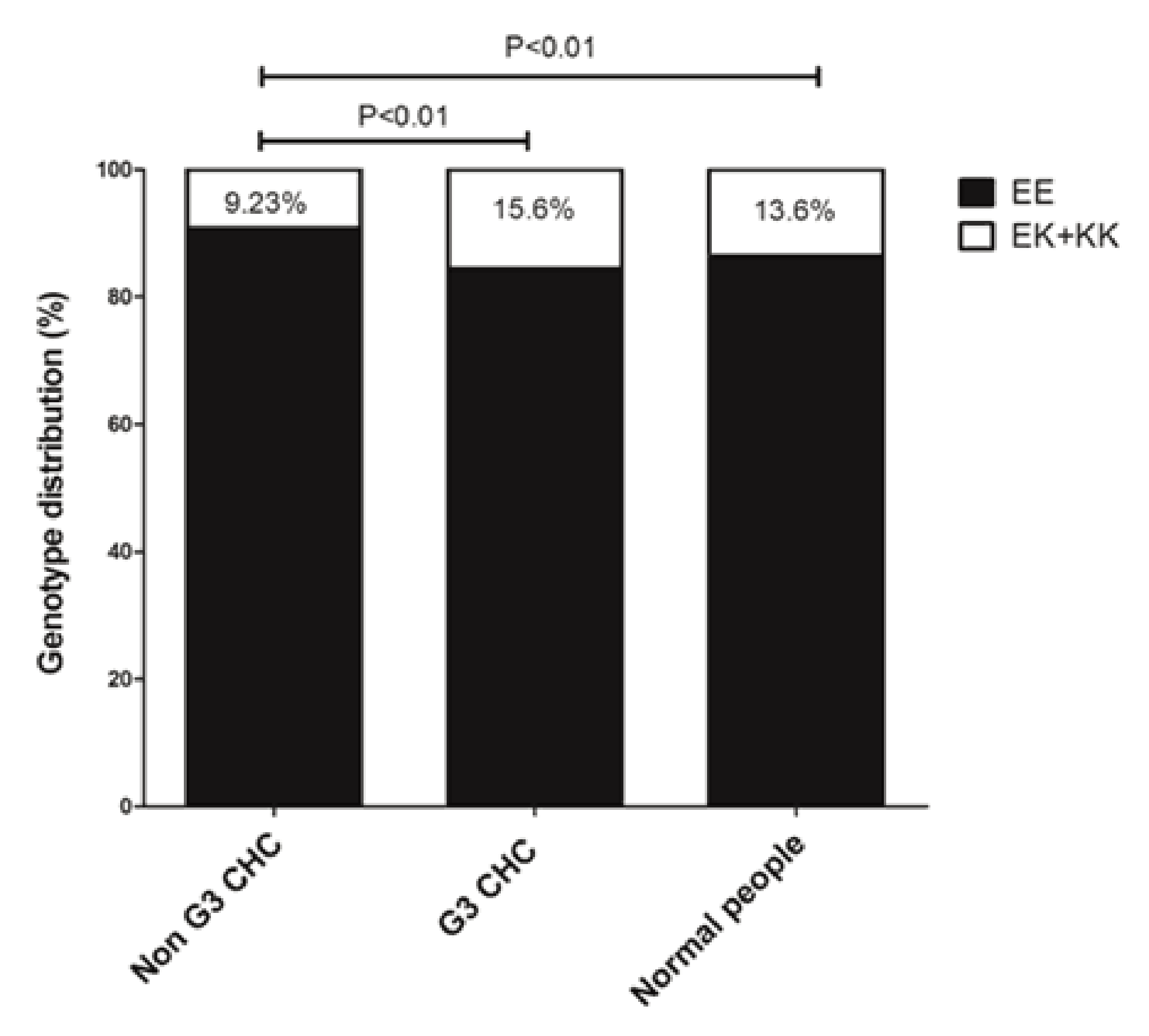


**Figure S2.** Association between the *TM6SF2* E167K variant and steatosis status in chronic hepatitis C patients

A. Pooled dichotomous odds ratio for *TM6SF2* E167K variant (EK+KK) on steatosis (≥S1) compared to non-steatosis group (S0) in chronic hepatitis C patients;

B. Pooled dichotomous odds ratio for *TM6SF2* E167K variant (EK+KK) on severe steatosis (S3) compared to non-steatosis group (S0) in chronic hepatitis C patients.


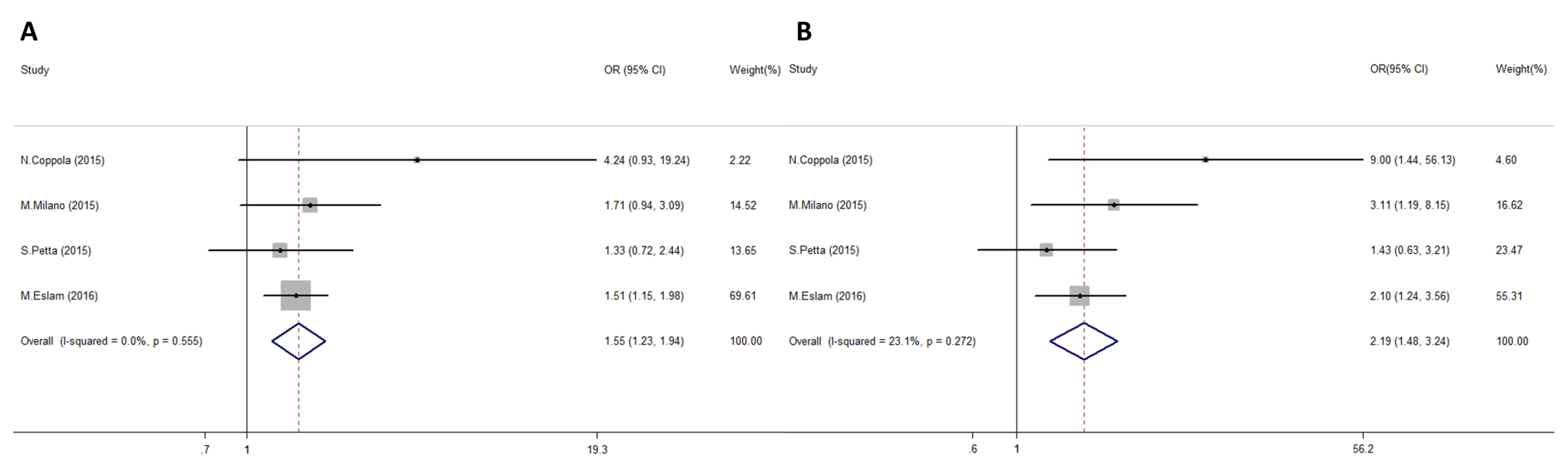


**Figure S3.** Sensitivity analysis on genetic impacts of *TM6SF2* E167K variant on histological features by stepwise omitting each study at a time

A. re-evaluation of continuous odds ratio of *TM6SF2* E167K variant (EK+KK) on steatosis severity after omitting each study;

B. re-evaluation of dichotomous odds ratio of *TM6SF2* E167K variant (EK+KK) on severe inflammation^a^ compared to non-severe inflammation group after omitting each study;

C. re-evaluation of dichotomous odds ratio of *TM6SF2* E167K variant (EK+KK) on significant fibrosis^b^ compared to non-significant fibrosis group after omitting each study.

^a^ severe inflammation was defined as G13-G18 status in study applying Ishak criteria [25] and G4 status in study applying Scheuer[26] and METAVIR [27] criteria;

^b^ significant fibrosis was defined as ≥F3 in studies applying for Ishak criteria [25], and ≥F2 in studies applying for METAVIR criteria [27].


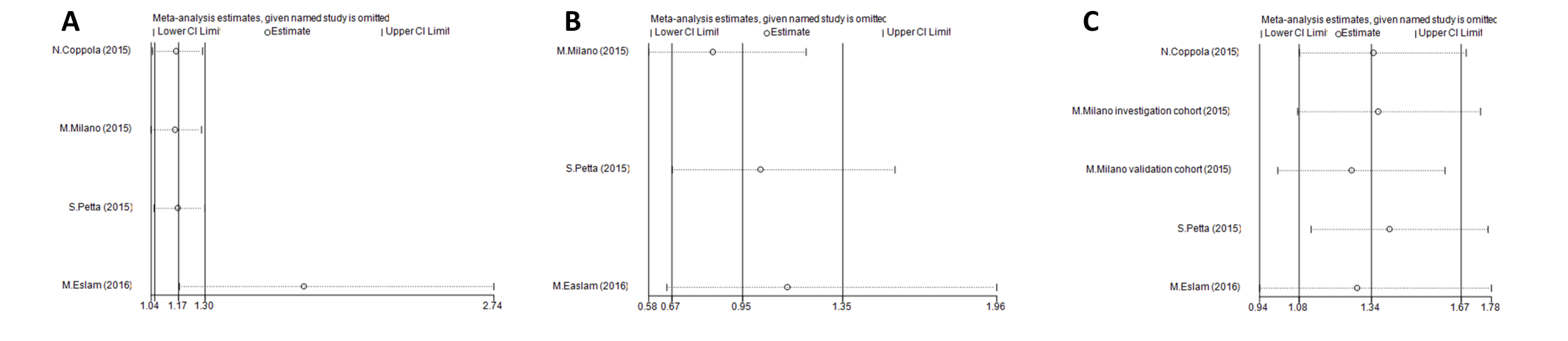


**Figure S4.** Cumulative meta-analysis of the genetic impact of the *TM6SF2* E167K variant on histological severity of liver damage in chronic hepatitis C patients

A. Cumulative pooled continuous odds ratio for T*M6SF2* E167K variant (EK+KK) on steatosis severity followed by enlarged sample sizes;

B. Cumulative pooled dichotomous odds ratio for *TM6SF2* E167K variant (EK+KK) on significant fibrosis^a^ compared to non-significant fibrosis group followed by enlarged sample sizes.

^a^ Clinically significant fibrosis was defined as ≥F3 in studies applying for Ishak criteria[25], and ≥F2 in studies applying for METAVIR criteria [27].


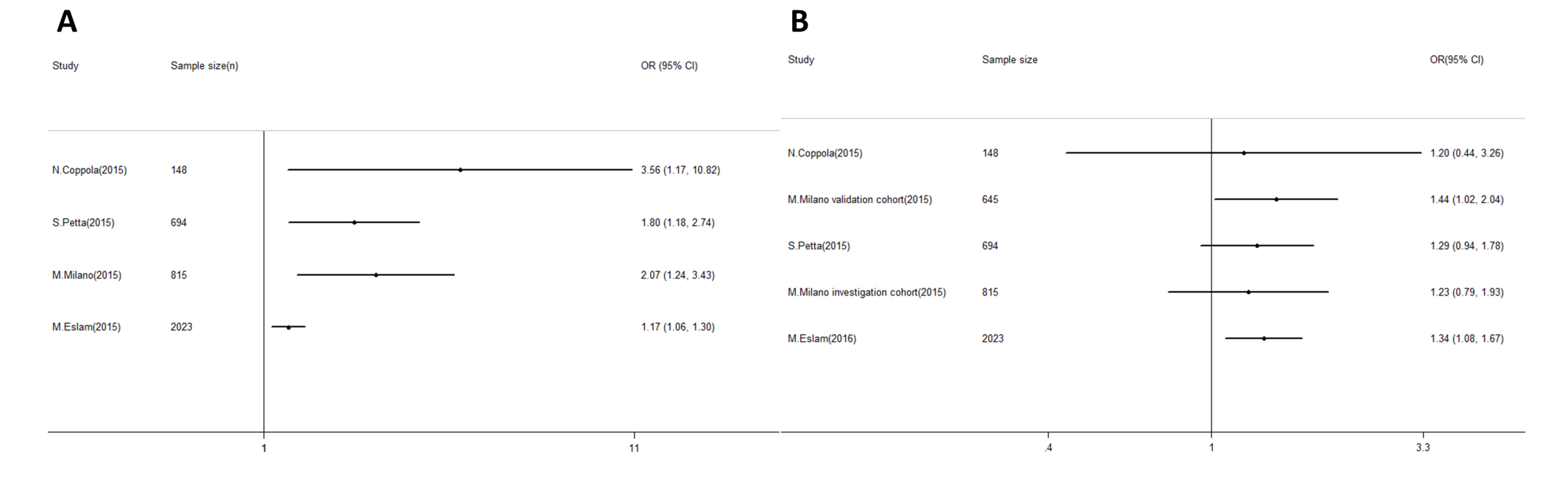


**Figure S5**. Association between TM6SF2 E167K variant and hepatic inflammation status in chronic hepatitis C patients

A. Pooled continuous odds ratio of TM6SF2 E167K variant (EK+KK) on inflammation severity in chronic hepatitis C patients;

B. Pooled dichotomous odds ratio for TM6SF2 E167K variant (EK+KK) on severe inflammation^a^ compared to mild and absent inflammation group in chronic hepatitis C patients.

^a^ severe/mild inflammation was defined as G13-G18/G0-G8 status in study applying Ishak criteria [25] and G4/G0-G1 status in study applying Scheuer[26] and METAVIR[27] criteria.


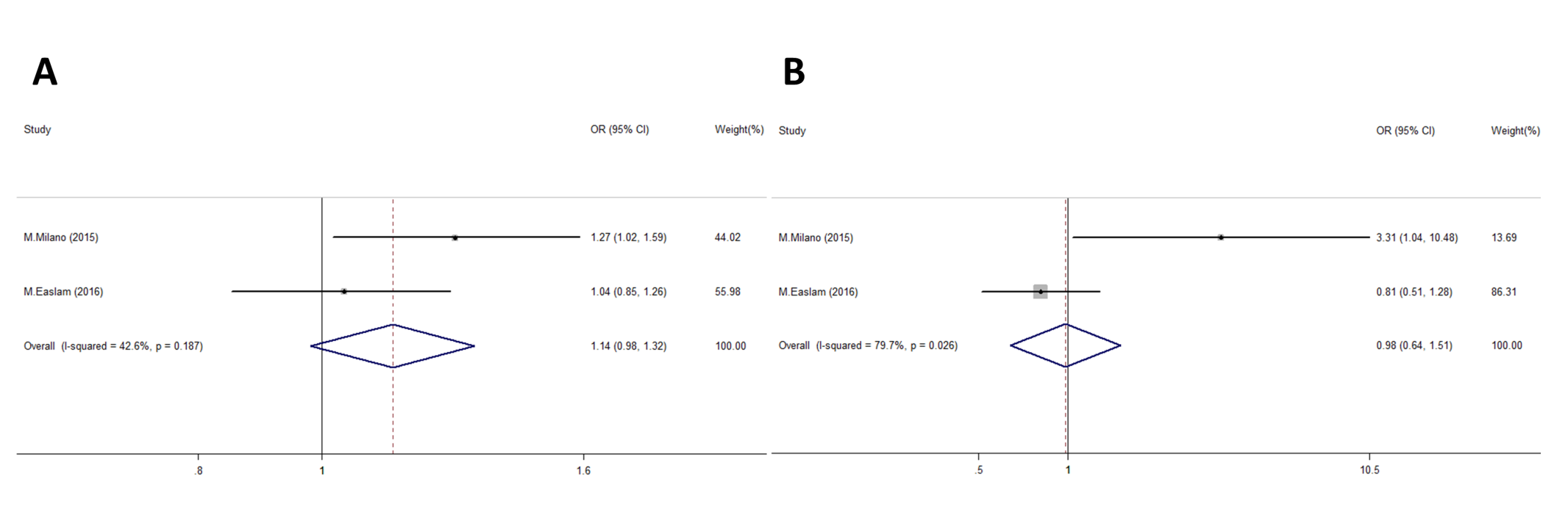


**Figure S6** Association between the TM6SF2 E167K variant and fibrosis status in chronic hepatitis C patients

Pooled dichotomous odds ratio of TM6SF2 E167K variant (EK+KK) on liver cirrhosis^a^ compared to non-cirrohosis group in chronic hepatitis C patients.

^a^ cirrohosis was defined as F6 status in study applying Ishak criteria[25] and F4 status in study applying METAVIR[27] criteria.


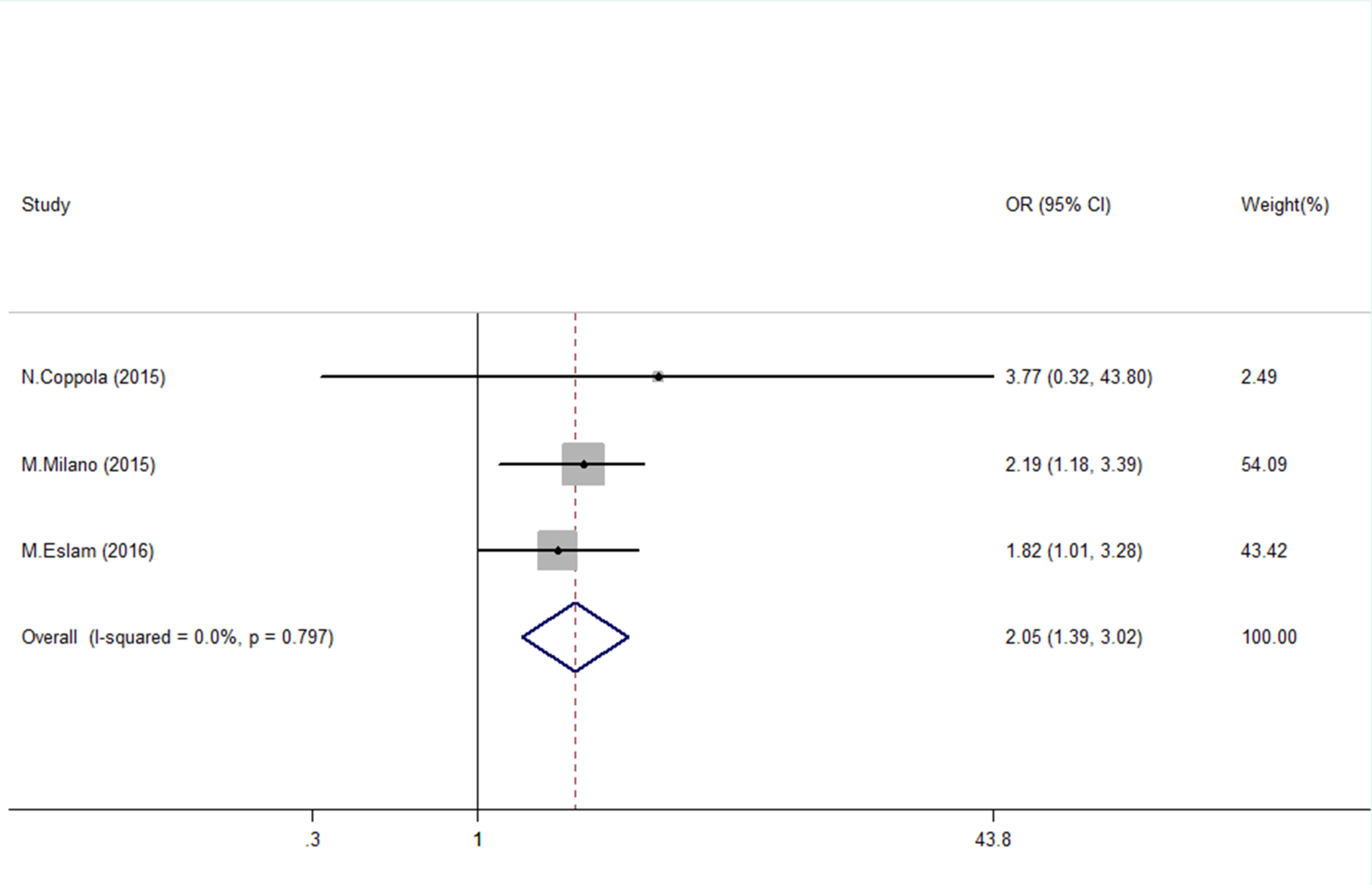


**Figure S7.**  Forest plot for genetic impact of *TM6SF2* E167K variant on fasting glucose level and insulin resistance in chronic hepatitis C patients

A. Pooled standardized mean differences of fasting blood glucose level in subgroups with different *TM6SF2* E167K genotypes (EK+KK vs. EE);

B. Pooled standardized mean differences of homeostasis model index in subgroups with different *TM6SF2* E167K polymorphism (EK+KK vs. EE).


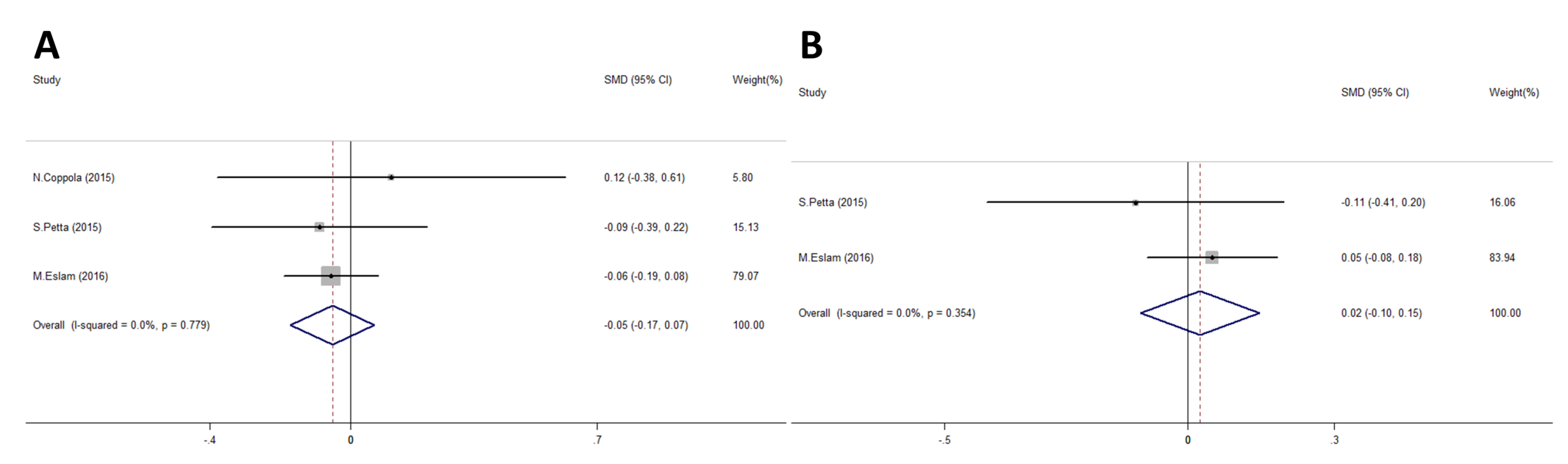


**Figure S8.** Forest plot for genetic impact of *TM6SF2* E167K polymorphism on body mass index in chronic hepatitis C patients.

Pooled standardized mean differences of body mass index in subgroups with different TM6SF2 E167K polymorphism (EK+KK vs. EE) categorized by study sample sizes.


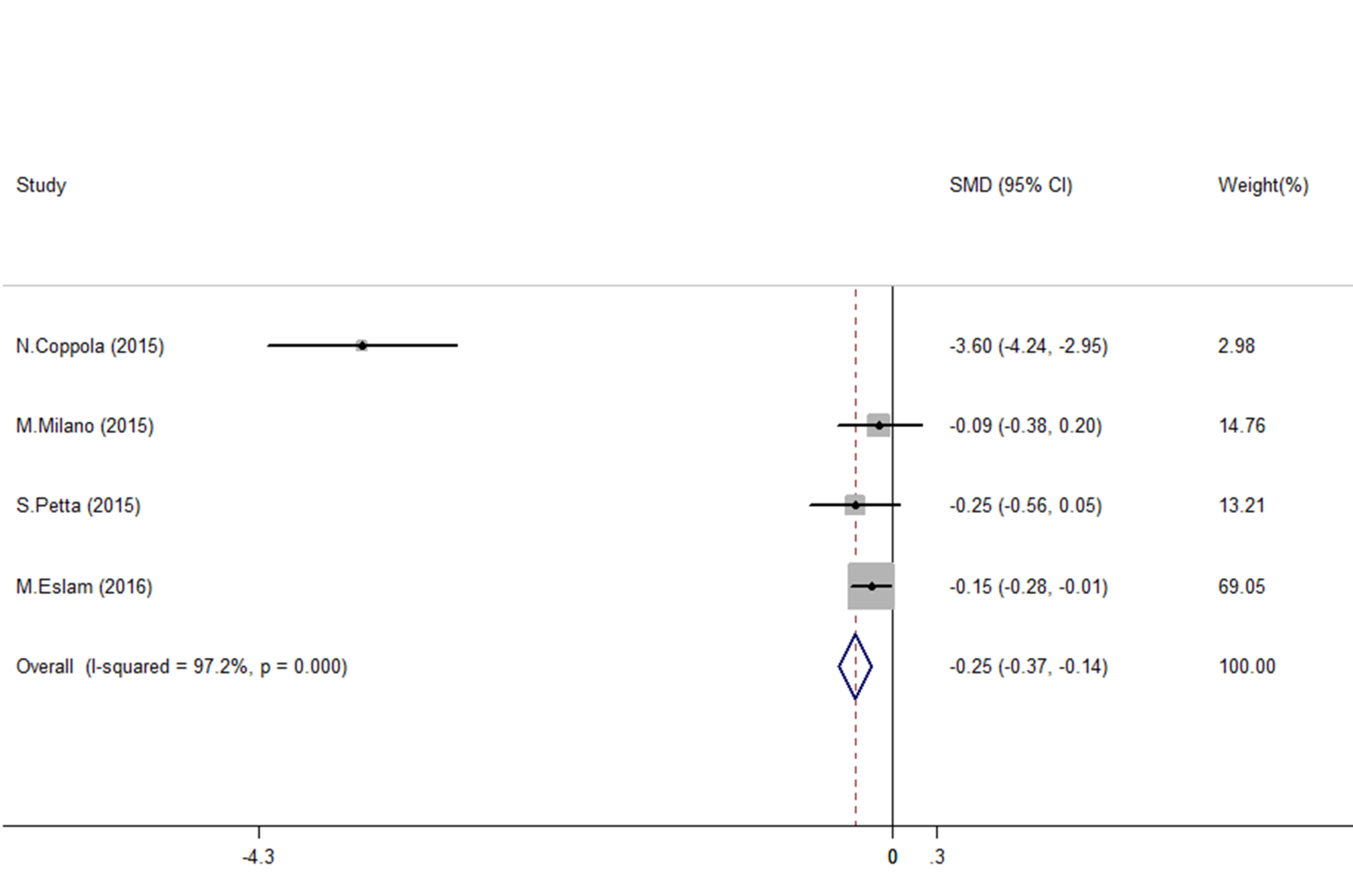


**Figure S9.** Forest plot for genetic impact of *TM6SF2* E167K polymorphism on log-transformed hepatitis C virus RNA level in chronic hepatitis C patients.

Pooled standardized mean differences of log-transformed RNA level in subgroups with different TM6SF2 E167K polymorphism (EK+KK vs. EE).


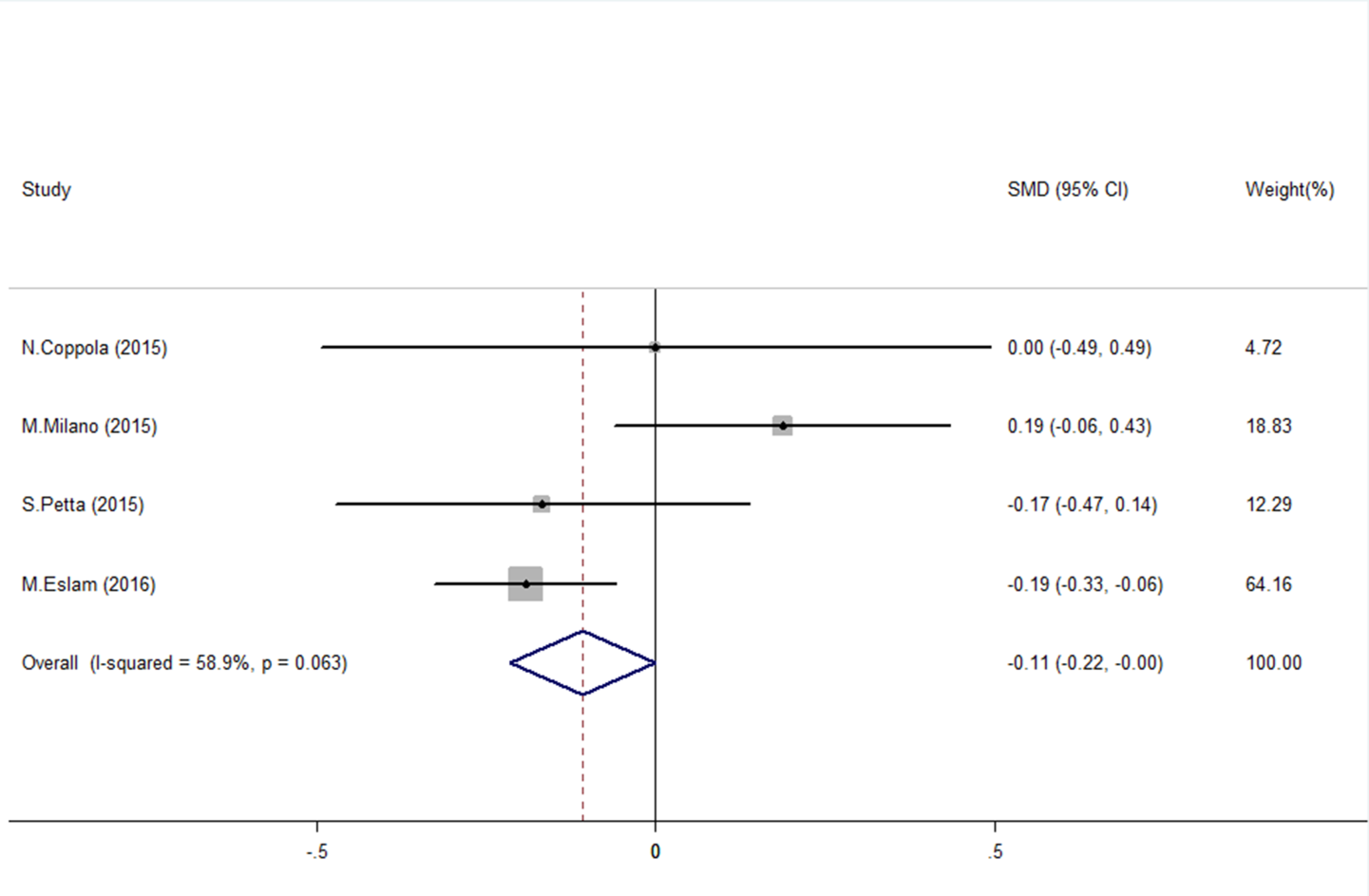


**Figure S10.** Epidemiologic model for the impact of lipid indicator on development of steatosis and fibrosis.

The phenotype-histological presentation association (dashed line) was evaluated based on genotype–phenotype and genotype-histological presentation associations (solid lines) from meta-analysis, using the Mendelian randomization approach. SMD and OR (in solid lines) were performed between subjects with variant and wild type TM6SF2 genotype (EK+KK vs. EE), respectively.


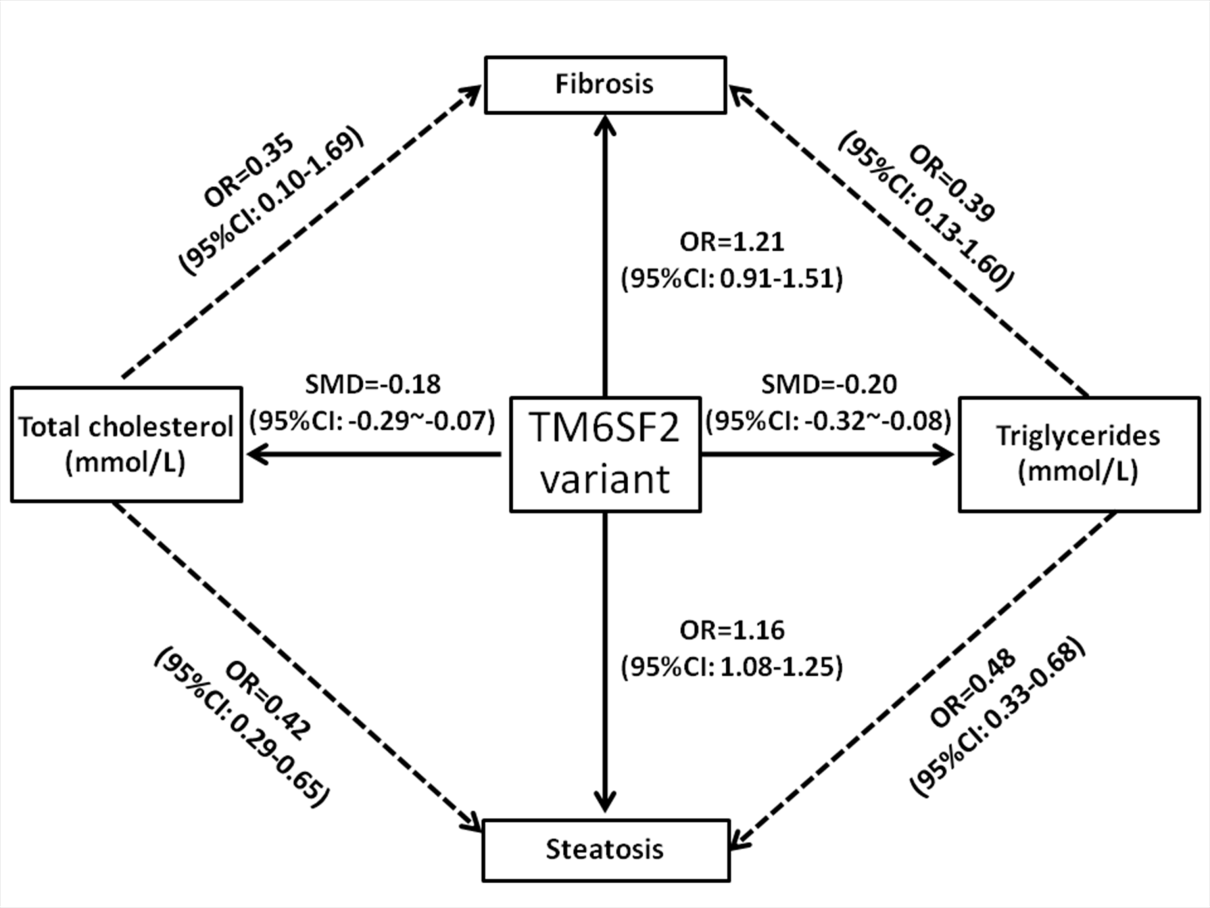


**Figure S11.** Begg’s funnel plot analysis of publication bias

A. Begg's funnel plot of publication bias for continuous odds ratio of TM6SF2 E167K variant (EK+KK) on steatosis severity, Egger's test: *P*=0.292, Bgger's test: *P*=0.174;

B. Begg's funnel plot of publication bias for dichotomous odds ratio of TM6SF2 E167K variant (EK+KK) on severe inflammation^a^ compared to non-severe inflammation group, Egger's test: *P*=0.298;

C. Begg's funnel plot of publication bias for dichotomous odds ratio of TM6SF2 E167K variant (EK+KK) on significant fibrosis^b^ compared to non-significant fibrosis group, Egger's test: *P*=0.161.

^a^ severe inflammation was defined as G13-G18 status in study applying Ishak criteria [25] and G4 status in study applying Scheuer[26] and METAVIR[27] criteria;

^b^ significant fibrosis was defined as ≥F3 in studies applying for Ishak criteria[25], and ≥F2 in studies applying for METAVIR criteria [27].


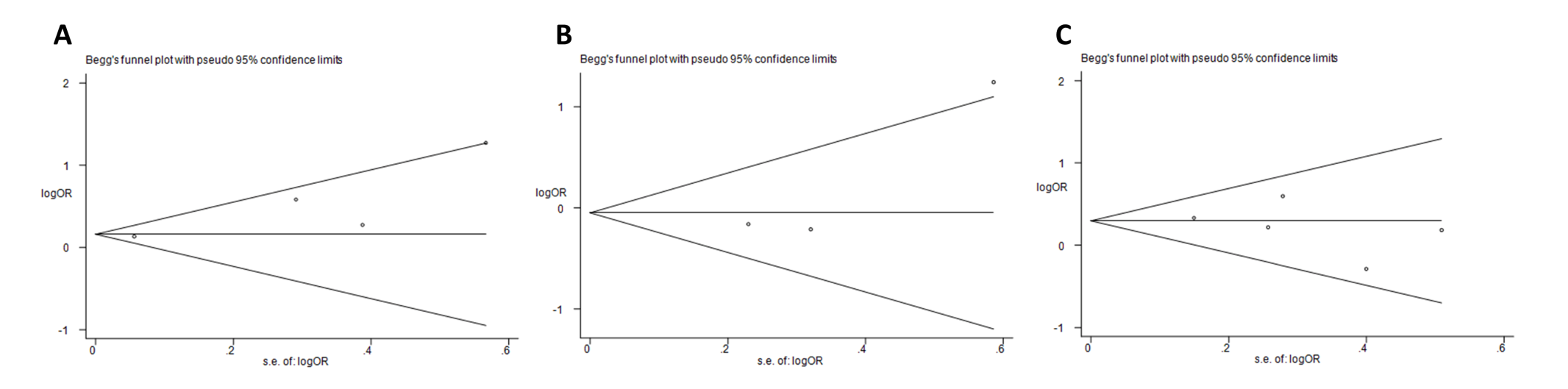

Supplement: Supplementary file 1 — Supplementary files [file 41598_2017_9548_MOESM1_ESM.doc]
